# Key Working for Families with Young Disabled Children

**DOI:** 10.1155/2011/397258

**Published:** 2011-07-21

**Authors:** Bernie Carter, Megan Thomas

**Affiliations:** ^1^School of Health, University of Central Lancashire, Preston PR1 2HE, UK; ^2^Children's Nursing Research Unit, Alder Hey Children's NHS Foundation Trust, Liverpool, L12 2AP, UK; ^3^Blenheim House Child Development and Family Support Centre, Blackpool Teaching Hospitals NHS Foundation Trust, Blackpool FY3 8LZ, UK

## Abstract

For families with a disabled child, the usual challenges of family life can be further complicated by the need to access a wide range of services provided by a plethora of professionals and agencies. Key working aims to support children and their families in navigating these complexities ensuring easy access to relevant, high quality, and coordinated care. The aim of this paper is to explore the key worker role in relation to “being a key worker” and “having a key worker”. The data within this paper draw on a larger evaluation study of the Blackpool Early Support Pilot Programme. The qualitative study used an appreciative and narrative approach and utilised mixed methods (interviews, surveys and a nominal group workshop). Data were collected from 43 participants (parents, key workers, and other stakeholders). All stakeholders who had been involved with the service were invited to participate. In the paper we present and discuss the ways in which key working made a difference to the lives of children and their families. We also consider how key working transformed the perspectives of the key workers creating a deeper and richer understanding of family lives and the ways in which other disciplines and agencies worked. Key working contributed to the shift to a much more family-centred approach, and enhanced communication and information sharing between professionals and agencies improved. This resulted in families feeling more informed. Key workers acted in an entrepreneurial fashion, forging new relationships with families and between families and other stakeholders. Parents of young disabled children and their service providers benefited from key working. Much of the benefit accrued came from strong, relational, and social-professional networking which facilitated the embedding of new ways of working into everyday practice. Using an appreciative inquiry approach provided an effective and relevant way of engaging with parents, professionals, and other stakeholders to explore what was working well with key working within an Early Support Pilot Programme.

## 1. Background

For families with a disabled child the usual challenges of family life can be further complicated by the fact that they often need access to a wide range of services provided by a wide range of professionals and agencies [1]. Whilst these services aim to provide the necessary medical, social, educational, and emotional support to the child and family, this can create a bewildering number of contacts and appointments for parents to manage, maintain, and integrate into family life. Families can feel lost in the system, unsure of what services are available to them, how to access them, and which professionals they should turn to for help in particular circumstances.

Recognition of these challenges for families has led to the implementation of family and child-centred initiatives such as Every Child Matters [2, 3], the Early Support Programme [4] and Aiming High for Disabled Children [1]. At national level, the Early Support programme is typical of these initiatives. It aims to ensure that services for young children with disabilities and their families across a range of sectors are based on partnership working “so that families are at the heart of discussion and decision-making about their children” and that service planning and delivery are integrated [5].

Common to most of these recent initiatives are effective multiagency working and key working in partnership with families. These two core strands, along with other improvements—such as enhancing responsiveness, timeliness, quality, and capacity of services—aim to ensure that the UK Government can fulfil its commitment that all disabled children should “have the best start in life and the ongoing support that they and their families need to fulfil their potential” [1]. 

The challenge for professionals is to ensure that families receive co-ordinated seamless services centred on parental choice and decision making [6] in which the difficulties of information sharing and “interpretation of confidentiality” [7, page 193], communication [8], and direction of focus [9] have been overcome. The benefits of working in partnership with parents are clear [10, 11]. Multiagency working can lead to positive results for children, families and staff [12–14]; Watson et al. [15, page 374] stress the “urgent need” to “work together”. 

Key working is core to most such initiatives as, in essence, it is

a way of managing the package of support available and ensuring families access the services to which they are entitled, with workers being named individuals who act as a single point of contact for multiple services, empower families and help them navigate the system [1, page 37].

In the same way that they act as a single point of contact for the families, key workers also act as a point of contact for the professionals and workers across a range of agencies. 

This paper is drawn from a larger study which aimed to evaluate what was working well within the Blackpool Early Support Pilot Programme (BESPP) which was one of the 45 Early Support Programme Pathfinder projects implemented by the UK Government as part of an initiative to improve the early support and care of disabled children.

## 2. Aims, Methodology, and Methods

The aim of this paper is to explore how the key worker role worked within the BESPP from the perspective of being a key worker and having a key worker.

### 2.1. Philosophical Approach

The main study used a narrative approach and drew on the principles of Appreciative Inquiry (AI) [16, 17] as we were concerned with identifying what was working well within the programme. Narrative and appreciative inquiry are both part of the qualitative research tradition. Using a narrative, discourse-based, relational approach provided a way of engaging with and developing analytical insight into the stories told by participants [18] as it is concerned with exploring the “complexities of the social world” [19].

The views of two, previously established, local Early Support (ES) steering groups were accessed to advise and guide the study design, suitability of questions, and alignment with the ethos of ES.

### 2.2. Methods

A mixed method approach was undertaken using (a) face-to-face surveys with parents, (b) e-survey of key workers and other stakeholders, (c) interviews with parents and key workers, and (d) an appreciative workshop (key workers, parents and stakeholders). All of the families on the pilot caseload (*n* = 10), their key workers (*n* = 8) and other stakeholders (from a diverse range of settings) were targeted. Data for this paper are drawn from all methods.

In essence, the face-to-face parental survey elicited families' experiences and perceptions from pre-referral to the BESPP through to assessment and diagnosis and beyond. The survey was undertaken by a representative from “Contact a Family” (a charitable organization for families).

The appreciative workshop drew on the principles of nominal group technique (NGT) [20] with the aims of generating group ideas and consensus. Participants were asked to focus on good practice and what they thought was working well. Their individual responses were then explored, challenged, and collated within a small group and then fed back to the main group for further discussion and the development of consensus statements.

Key workers' and other stakeholders' perspectives on the service and their role within it were accessed through a survey using open and closed questions that were sent to them by email. 

The caseload parents and key workers (not matched dyads/triads) were invited to participate in an appreciative, narrative face-to-face interview with the lead researcher in either their home or work setting. The aim was to build a picture of participants' experiences with ES and to elicit further specific evidence of how the pilot had contributed to the lives of the children and their families.

### 2.3. Ethics and Research Governance

Ethics approval was gained from the Faculty of Health Ethics Committee at the University of Central Lancashire. Prior to this, the proposal had been presented to the Chair of the Local Research Ethics Committee (LREC) for consideration but it was not deemed necessary to submit it for LREC approval.

## 3. Data Analysis

All qualitative data were transcribed and subjected to detailed line-by-line coding; the codes were then collapsed into themes and core themes. Data analysis (supported by Atlas.ti) was systematic, with each participant's contribution being considered individually as well as part of a whole. The themes were then reexamined to locate the core stories which illustrated the experiences of, insights into, and the impact of the pilot. The findings are presented as groups of stories/themes with illustrative quotations. Abbreviations denote the source (P: parent, KW: key worker, OS: other stakeholder).

The sample consisted of

- 8 (of 10) caseload families participated in the face-to-face surveys; 2 also participated in the appreciative interviews (5 girls, 3 boys);
- 6 (of 8) key workers (6 in the survey, 3 were also interviewed and 2 also contributed to the workshop);
- 29 key stakeholders (representing education, social care, acute health care, community-based health care, and voluntary care).

## 4. Findings and Discussion

In this paper, we focus on the narrative findings about “what was working well” in relation to key workers and key working. These findings are discussed, where relevant, in the context of the changes brought about through the BESPP. Four major findings—“a central and collaborative intelligence,” “having a key worker,” “being a key worker,” and “developing key working skills”—were identified. The discussion of these findings and their integration with key literature are presented coterminously. 

The key workers' backgrounds were diverse and included portage home visitors (visitors with specific training who support parents through suggesting activities and routines to encourage their child's development) as well as specialist health visitors. The key workers were given remission from their main professional duties to undertake their key working role. This inevitably involved juggling commitments, and invariably the key workers would have valued more dedicated key working time especially in the early days.

### 4.1. Central and Collaborative Intelligence

In essence, the key workers saw their role, as did the parents and other stakeholders, as the person whose responsibility was to coordinate, manage, and have a professional overview of a family's ES needs. This depiction is similar to the ways which key working has been portrayed by key workers and parents in other studies [21–24]. One stakeholder synthesized their role as being the “central intelligence” of service delivery and this concept was instantly recognized by the key workers, families, and stakeholders when we took it back to them for authentication. However, a word of caution here; the key workers did not hold a monopoly over this information. In reality, this intelligence was family-led, shared, dynamic, and collaborative. It resulted in the needs being identified, and interventions and assessments fitting together so as to try and ensure that the child had the “best possible start and best possible care” (KW). In essence, the key workers acted a central (professional) repository of knowledge which reciprocally complimented the family's repository of knowledge about their child. The key workers acted as an access point for this knowledge within an overtly “family and needs led” service. They shared their understanding of it, as appropriate, with other professionals on the families' behalf, helping to facilitate actions which were sensitive to “individual family needs” (P) occurring at the “right time for parents” (P). 

The ability to forge this mutual and shared intelligence was supported by the BESPP's new processes and protocols which meant that everyone involved had “clear … guidelines” (OS) to work within making it easier to agree and “reach decisions” (KW). Clearer protocols meant that written information from meetings, assessments and interventions was documented in a way that was accessible and available to parents as well as professionals thus increasing parents' satisfaction with information. Stewart et al.'s [25, page 498] study notes how “information becomes a powerful tool for supporting children and ensuring an equal voice for parents” and the Parent Information KIT they developed resulted in enhancing parents' ability and self-confidence in “getting, giving and using information to assist their child with a disability.” As with other studies, we saw evidence of the importance of clear, jointly negotiated protocols to support the development of best practice [26].

### 4.2. Having a Key Worker

Everything has gone really well …  it's all been positive from day one. It has been a really good experience to be honest. Everybody has been so helpful. My key worker is absolutely fantastic, I think I would have gone mad without her. They're all great people. It makes you feel a lot easier when you know that they are at the end of the phone and that they will come out. It takes a lot of pressure and stress off the family so that you can get on with day-to-day things (Mother).

Having a key worker meant that the parents were clearer about who was responsible for specific aspects of their child's care and everyone started to see the “whole picture instead of just their piece of the jigsaw” (OS). Most of the mothers described the relief of not “having to do it all on my own” and “just being able to pick the phone up and know that the key worker will sort it out for you”. Many of the differences the parents experienced were based on the bond the key workers had with them and their children, the mutual trust that developed, and the emotional support they provided to the family. 

Listening to the parents' descriptions of the key workers in action brought to mind descriptions of superheroes with a range of superpowers helping families tackle the problems they faced. However, despite being “brilliant” (P) and “wonderful” (P) the key workers had no special powers and relied, in their own words, on “common sense,” “dogged persistence,” “a sense of humour,” and “being just plain stubborn” (KW) to get results. 

Simply put, the key workers gave mothers more time to “get on with being a mum” (P) by making life more manageable and taking away much of the burden of having to integrate the administration of the tasks associated with appointments, treatments, professionals, and hospital visits into family life. They had the ability to “bust through the bureaucracy” and “take away the mundane jobs” that the parents had previously had to do such as “making a 101 phone calls for one appointment or to get a prescription changed” (P). Having a key worker provided parents with both emotional support and a proactive professional they could trust to help them find solutions to problems and who had real insight into their needs as a family and who was committed to work with and for them. As Fereday et al. [27, page 629] propose, the parents in our study wanted a “partnership relationship, grounded in respect, sensitivity and understanding of their child's needs and the wider orbit of their family life.” These relatively modest, albeit challenging, demands were ones which the key workers saw as essential elements to the way that they worked with their families.

The key workers' sensitivity to family life meant that they appreciated the drain on family resources that multiple appointments created. Trusting families' expertise and insight to know which appointments were important and which were “unnecessary check-ups” where you “just turn up to a meeting every six months to prove you (are) a good girl” (P), the key workers were able to help families rationalize and coordinate appointments. The parents wanted this not only because it reduced the demands on their own individual families but also because they understood that services and resources were stretched and wanted to contribute to effective service use by helping to free up services and resources for other parents. 

Due to the reciprocal and collegial approach adopted by the key workers, families felt secure in discussing issues and concerns as well as successes and achievements with them. This fostered mutual understanding of those “little things that you're not sure whether you should bother people with” (P) and meant that unmet needs could be more easily identified and then addressed by the most appropriate professional(s). 

The benefits the parents experienced were often relational ones. The key workers ensured that parents knew the people caring for their child, felt they were involved, informed and their parental expertise valued, and felt able and welcome to contribute. One mother explained the benefits when she said “I don't think we've ever felt like we didn't know what was going to happen.”

Their high level of engagement with families and the fact they did not take control away from the parents meant that, unlike the families in Case's [28] study, they were not marginalised or disempowered by “expert” professionals. Instead, the key workers' use of what Blue-Banning et al. [29] describe as facilitative behaviours (communication, commitment, equality, skills, trust, and respect) supported equitable partnerships.

Key workers acted as an information conduit, guiding parents to appropriate sources of information and other resources as has been shown in other studies [30]. Whilst less sophisticated than the information management system described by Stewart et al., [25] they did assist parents to manage information. They also provided “welcome support” by helping parents “fazed” by the paperwork associated with having a child with complex needs/disabilities to navigate their way through the bureaucracy and as one mother explained:

… and with the family meetings we'll sit down and I'll say, ‘I really don't know where to start or what I want to discuss'! So without her it'd have been just a blank sheet and not an agenda! But she'd say things like ‘Why don't we try and start to go for this or maybe think about that' …. So having her there as a support, as someone I can pick the phone up and talk about a meeting that's coming up has been really positive. We've all–all the family–have got on really brilliantly with her. She's been really great (Mother).

Although parents were glad to attend and contribute to meetings about their child, they were very grateful that the key workers did “all the minutes and all the reports” (P).

Key working also brought major benefits for other professionals who no longer had to chase “different things that weren't part of their role” (KW).

### 4.3. Being a Key Worker: Becoming Entrepreneurial

But I think the families have really gained from having a key worker, and it's not just because it's been me personally, but they've actually gained because the professionals have wrapped round them rather than the parents have had to go and talk to people at different times (Key Worker).

The early months of key working were challenging and for some of the key workers, establishing the role was “not easy at all” (KW) and the “learning curve [was] quite steep.” The key workers' narratives emphasized the ways in which they had to develop their own knowledge, networks, and understandings about the “nitty-gritty” of providing support. Whilst they may have been operating at expert level within their own professional domain, some found the initial move into key working challenging. They had to identify what services were available, the routes to accessing services, who to contact and how to contact them, and ways of working with, around or subverting systems. To some degree, the key workers experienced the same sort of challenges that parents (without a key worker) face when trying to negotiate a large, complex,and often obscure bureaucracy. 

The key workers had to be highly flexible, adaptive and entrepreneurial. Describing themselves as “forging the way and sorting things out” they felt supported in their role by the parents who they felt were behind them “all the way.” As one key worker said “when you're working so closely with a family, failure is just not an option.” They talked of their role in terms of “being there (for the family),” “being persistent,” “being determined,” “finding ways round obstacles,” “finding answers,” “looking for information,” “making connections,” “being an advocate”, “being at the centre of a hub,” and “being the point of contact for the family” (KW). These attributes are broadly features of an entrepreneurial mindset which is characterized by seeking and pursuing new opportunities and innovation, accepting risks, being adaptive, focusing on (adaptive) execution, creating and sustaining networks of relationships, and “engaging the energies of everyone in their domain” [31, page 2]. 

Sharing information was sometimes “tricky,” requiring an appreciation of a range of different perspectives and vested interests. Information-sharing required trust, willingness, and insight into the roles and responsibilities of the different professionals. Whilst some stakeholders thought key workers should adopt an ultra cautious approach to information-sharing due to concerns about breaching confidentiality, the parents in our study trusted their key workers to share information appropriately. They balanced any potential risks against the benefits of better informed staff, reduced need to repeat basic information, and smoother movement through services. Dalzell et al. [9,page 582] also noted that families were “happy to share their information with other professionals,” and in Gray et al.'s [32] study “confidentiality smokescreens” were overcome as confidence in Multiagency working increased. 

Although the key worker role created, in most cases, a lot of additional work for the individual (including more paperwork) they valued the role and “learned a lot”. It also widened the individual key workers' horizons, giving them the opportunity to move away from being “cocooned” (KW) in their own potentially narrow disciplinary world. The key workers in our study gained increased job satisfaction as also demonstrated in other studies [24, 33].

### 4.4. Developing Key Working Skills

Developing uncertainly at first but then with increasing confidence, the key workers became what Atkinson et al. [34,page 225] describe as a “new and “hybrid” professional type who has personal experience and knowledge of other agencies, including, importantly, these services' cultures, structures, discourse, and priorities. 

Their starting point was their own disciplinary expertise and the key worker training they had undertaken before commencing their role. However, their skills and abilities developed exponentially with the experiential learning that occurred within their day-to-day practice with families and other professionals and agencies. Indeed, the diversity of disciplinary background meant that as a group they had different educational, professional, and philosophical starting points and this added richness to the experience, knowledge and approaches of the key workers. As one key worker who divided her time between her own practice and her key working described:

Based here (place of work) in the speciality I'm working in I don't do any home visits and I'm cocooned in my own little world here. So being a key worker I'm out and about a lot more. I meet a lot more professionals that I wouldn't necessarily normally come into contact with and … visiting the family at home. And really that opened my eyes to a lot of things that I'd not really seen before and just really building up a relationship with the family that I don't think I could have had just through working here. I think as a key worker that changed my perception really.

This reflexive, flexible approach was evident in all the key workers' stories and, as in White and Featherstone's [8] study, our key workers benefited from exposure to other disciplines and they modelled what key working was and could be. One of the early and ongoing challenges was creating a shared understanding of the role:

(Key working's) not very easy I don't think, not with the people who aren't directly involved in it. And I think you can't just say “key workers” to parents and other people working with families, because [key working] means different things to different people. So in that sense it isn't that easy and I don't think it can be because there's all the different descriptions of it everywhere, they need to be interested to look at the information to see exactly what it means. So that's never going to get easier until it's common practice basically (Key Worker).

An unexpected skill many of the key workers developed was that of being a “detective” as

… every family situation is so different and diverse. There's always more information to find out … it's endless really, the things that you need to know! There's key things, key resources for families—they're not so hard …. But you're always needing to pick up things you've never heard of before, you're always looking around for sources of information and help. And you're always coming across something that you didn't know about. Although you'd go to key agencies for some things there are others that are harder to find …. I suppose it's like doing a bit of detective work sometimes to find out where that particular resource comes from

Questions posed by the parents or resources needed by the child often resulted in some high level detective work being required to track down some specific information, the right person, for the job or the “right string to pull.” This knowledge was acquired with various degrees of ease and difficulty. As one key worker noted “It's hard enough for us… it must be awful if you're the parent.” The challenges of key working meant that the key workers really appreciated the uphill struggle that parents would face if they had to do this for themselves. Indeed when admitting the challenge of fitting key working into their existing role, they acknowledged the difficulties that parents, managing without a key worker, faced in addition to their parental role.

All of the key workers reported enjoying the role, finding it stimulating, and helping them to develop new or enhance existing skills. Key working was a stressful, major undertaking, eating significantly into their other work time as it meant “… being asked to do extra, extra” (KW). Similar problematic time constraints are evident in other studies [22, 34]. Disparities existed between key working with different families as well as the different stages in an individual family's support journey. Beecham et al. [35, page 617] note that “other things being equal, children with higher levels of disability may well require higher than average levels of support (higher costs).”

## 5. Conclusion

The key workers were instrumental in enabling children, and their families to access services and, as such, this was promoting the Government's aspiration of supporting disabled children, young people and their families “to play a full role in the society of which they are part, and will benefit from equality of opportunity compared to their peers” [1, page 6].

As Greco and Sloper [23, page 14] note the “simplicity of the idea [key working] is contrasted by the complexity of its implementation.” However, despite the complexity, the benefits of key working were real and as seen in other studies the key workers did make a real difference to everyday lives of children and families [35]. Key workers became both adept at “letting go of their habits … (and) … understanding the “rationalities” of other professions …” [8, page 215] as well as providing support to parents who were able to take control of decision-making in relation to their child's life.
